# Rapid Identification of Seven Waterborne *Exophiala* Species by RCA DNA Padlock Probes

**DOI:** 10.1007/s11046-018-0256-7

**Published:** 2018-03-05

**Authors:** M. J. Najafzadeh, V. A. Vicente, Peiying Feng, A. Naseri, Jiufeng Sun, A. Rezaei-Matehkolaei, G. S. de Hoog

**Affiliations:** 1 Department of Parasitology and Mycology, Faculty of Medicine, Mashhad University of Medical Sciences, Mashhad, Iran; 2 Cancer Molecular Pathology Research Center, Mashhad University of Medical Sciences, Mashhad, Iran; 3 Microbiology, Parasitology and Pathology Post-Graduation Program, Department of Basic Pathology, Federal University of Paraná, Curitiba, Brazil; 4 Third Affiliated Hospital, Sun Yat-sen University, Guangzhou, China; 5 Guangdong Provincial Institute of Public Health, Guangdong Provincial Center for Disease Control and Prevention, Guangzhou, China; 6 Department of Medical Mycology, School of Medicine, Ahvaz Jundishapur University of Medical Sciences, Ahvaz, Iran; 7 Westerdijk Fungal Biodiversity Institute, Utrecht, The Netherlands; 8 Center of Expertise in Mycology Radboudumc/Canisius Wilhelmina Hospital, Nijmegen, The Netherlands

**Keywords:** Waterborne *Exophiala*, Black yeasts, Identification, Rolling circle amplification

## Abstract

The black yeast genus *Exophiala* includes numerous potential opportunistic species that potentially cause systematic and disseminated infections in immunocompetent individuals. Species causing systemic disease have ability to grow at 37–40 °C, while others consistently lack thermotolerance and are involved in diseases of cold-blooded, waterborne vertebrates and occasionally invertebrates. We explain a fast and sensitive assay for recognition and identification of waterborne *Exophiala* species without sequencing. The ITS rDNA region of seven *Exophiala* species (*E. equina, E. salmonis*, *E. opportunistica, E. pisciphila, E. aquamarina, E. angulospora* and *E. castellanii*) along with the close relative *Veronaea botryosa* was sequenced and aligned for the design of specific padlock probes for the detection of characteristic single-nucleotide polymorphisms. The assay demonstrated to successfully amplify DNA of target fungi, allowing detection at the species level. Amplification products were visualized on 1% agarose gels to confirm specificity of probe–template binding. Amounts of reagents were reduced to prevent the generation of false positive results. The simplicity, tenderness, robustness and low expenses provide padlock probe assay (RCA) a definite place as a very practical method among isothermal approaches for DNA diagnostics.

## Introduction

*Exophiala* is a member of the ascomycete order *Chaetothyriales* (fungi), comprising the black yeasts and allies, which are frequently encountered as causative agents of disorders in humans and animals [1–7]. Human infections vary from commensalism or moderate cutaneous infection to fatal neurotropism with serious mutilation. Infections frequently involve patients without known immune disorder or underlying metabolic disease. Outside humans, especially cold-blooded waterborne vertebrates are susceptible to a variety of *Exophiala* species [2], some of these seem to be specific to certain host taxa [1]. As virulence factors, the capability to absorb alkylbenzenes, within sweat and nervous tissues of mammals and in the poisonous skin of amphibians, has been proposed [2, 8, 9]. Studies on epizootics from the older literature obviously show that black yeast infection is a relatively popular phenomenon in cold-blooded vertebrates [10–15]. Recent molecular reports demonstrate that various pathogenic species are concerned [1, 2, 16], which morphologically are extremely similar.

Some species can be classified by physiological characteristics, for example, temperature tolerance and nitrate assimilation, however, for many taxa molecular characterization are needed. [17]. Sequencing of the rRNA ITS location is generally adequate for routine species distinction in the genus *Exophiala* [18]. This method is relatively costly and time-consuming and less suitable for large numbers of strains in case of monitoring of epizootics.

Rolling circle amplification (RCA) is an isothermal DNA amplification technique applying so-called padlock probes. The method has been proven to be fast, cost-effective and specific for identification of human and plant pathogenic fungi [6, 19–25], including black yeasts and relatives [26–28]. The 3′- and 5′-end strands of the probes hybridize next to one another at the target strand, leading to circularization of the molecule upon ligation. The circular molecule is consequently amplified isothermally with a DNA polymerase that lacks exonuclease activity, and the resulting product subsequently can be utilized with a second primer causing a cascade of amplifications. Because of the necessary accurate base pairing, the padlock probes have the ability to identify single position mutations [29–31].

In the present paper, we developed eight padlock probes on the basis of the ITS location to identify the most relevant species of *Exophiala* in animal infection and epizootics, viz. *E. equina, E. salmonis*, *E. opportunistica, E. aquamarina, E. angulospora* and *E. castellanii,* together with *Veronaea botryosa* as out-group. The objective of the current study was to evaluate the practical applicability of the method and to assess its limitations.

## Materials and Methods

### Strains

The 62 isolates of *Exophiala* and *Veronaea* included 13 strains of *E. equina*, 3 of *E. salmonis*, 6 of *E. opportunistica,* 6 of *E. pisciphila,* 8 of *E. aquamarina,* 10 of *E. angulospora,* 7 of *E. castellanii* and 9 of *V. botryosa* (Table 1); together these strains formed the ‘salmonis-clade’ of waterborne mesophilic species [2]. Isolates originated from cold-blooded animals, from human infections and from the environment. Cultures are preserved on slants of 2% malt extract agar (MEA) and oatmeal agar (OA) (Difco, Brunschwig, Amsterdam, the Netherlands) at 24 °C in the reference collection of the Centraalbureau voor Schimmelcultures (housed at Westerdijk Fungal Biodiversity Institute, Utrecht, the Netherlands). Affiliation to *Exophiala* was verified using a phylogenetic tree constructed with sequences of the partial SSU gene. Species identity was confirmed by sequencing rDNA internal transcribed spacer (ITS), partial β-tubulin (*BT2*), partial elongation factor 1-α (*TEF1*) and actin (*ACT1*) genes [2]. To evaluate the specificity of padlock probes, we tested four closely related species: *Cladophialophora bantiana* (CBS 678.79), *Exophiala dermatitidis* (CBS 525.76), *E. bergeri* (CBS 526.76) and *Rhinocladiella mackenziei* (CBS 650.93).

**Table 1:** Strains analyzed

Name	Number	Source	Geography	GenBank (ITS)
*E. equina*	CBS 109789	Human, dialysis	The Netherlands	JF747086
*E. equina*	CBS 115143	Bottled water	Australia	JF747080
*E. equina*	CBS 120906	Stool	USA	JF747093
*E. equina*	CBS 120905	Human, ulcer cornea	The Netherlands	JF747088
*E. equina*	CBS 120904	Water from water machine	The Netherlands	JF747081
*E. equina*	CBS 121285	Human, skin flakes	The Netherlands	JF747090
*E. equina*	CBS 121282	Human	USA	JF747091
*E. equina*	CBS 121501	Drinking water	The Netherlands	JF747077
*E. equina*	CBS 121513	Water system of packaging machine	The Netherlands	JF747082
*E. equina*	CBS 116009	Galapagos turtle	USA	JF747095
*E. equina*	CBS 116922	Silica gel	The Netherlands	JF747097
*E. equina*	CBS 109913	Drinking water	Germany	JF747145
*E. equina*	CBS 150,93	Washed Tilia root	Germany	JF747096
*E. salmonis*	CBS 110371	Frog	USA	…
*E. salmonis*	CBS 157,67	Trout, brain	Canada	JF747137
*E. salmonis*	CBS 120274	Drinking water tap	The Netherlands	JF747138
*E. opportunistica*	CBS 631,69	Unknown	The Netherlands	JF747128
*E. opportunistica*	CBS 122268	Human, foot	Denmark	JF747125
*E. opportunistica*	CBS 660,76	Rhizosphere, *Triticum aestivum*	West Australia	JF747126
*E. opportunistica*	CBS 122269	Human, nail	Denmark	JF747124
*E. opportunistica*	CBS 637,69	Polyvinyl alcohol	Unknown	JF747127
*E. opportunistica*	CBS 109811	Drinking water	Germany	JF747123
*E. pisciphila*	CBS 119913	Potbelly seahorse	Unknown	JF747132
*E. pisciphila*	CBS 119914	Potbelly seahorse	Unknown	JF747133
*E. pisciphila*	CBS 121500	Human, nail	Germany	JF747134
*E. pisciphila*	CBS 101610	Water pipe	Germany	JF747130
*E. pisciphila*	CBS 121505	Swimming pool	Germany	JF747129
*E. pisciphila*	CBS 537,73	Catfish	USA	JF747131
*E. aquamarina*	CBS 119915	Little tunnyfish	USA	JF747061
*E. aquamarina*	CBS 120417	Leafy seadragon, bone	USA	JF747057
*E. aquamarina*	CBS 119919	Leafy seadragon, skull	USA	JF747056
*E. aquamarina*	CBS 119912	Winter flounder	USA	JF747060
*E. aquamarina*	CBS 119921	Weedy seadragon	USA	JF747059
*E. aquamarina*	CBS 119916	Leafy seadragon, necrotic tissue	USA	JF747055
*E. aquamarina*	CBS 119917	Leafy seadragon	USA	JF747058
*E. aquamarina*	CBS 119918	Leafy seadragon, skin	USA	JF747054
*V. botryosa*	CBS 121506	Human, wrist skin	Japan	JF747140
*V. botryosa*	CBS 122826	Railway tie	Brazil	…
*V. botryosa*	CBS 122236	Railway tie	Brazil	…
*V. botryosa*	CBS 122823	Railway tie	Brazil	…
*V. botryosa*	CBS 122824	Railway tie	Brazil	…
*V. botryosa*	CBS 122825	Railway tie	Brazil	…
*V. botryosa*	CBS 102593	Human, disseminated in child	China	JF747142
*V. botryosa*	CBS 101462	Human, skin	Unknown	JF747141
*V. botryosa*	CBS 254,57	Sansa olive slag	Italy	JF747143
*E. angulospora*	CBS 119911	Weedy seadragon	USA	JF747050
*E. angulospora*	CBS 122237	Hydrocarbon polluted soil	Brazil	…
*E. angulospora*	CBS 120272	Drinking water tap	The Netherlands	JF747045
*E. angulospora*	CBS 121503	Fish	Russia	JF747049
*E. angulospora*	CBS 122264	Human, leg	Denmark	JF747052
*E. angulospora*	CBS 146,93	Tilia wood	Germany	JF747053
*E. angulospora*	CBS 441,92	Man, nail	Netherland	…
*E. angulospora*	CBS 617,96	Wood	New Zealand	JF747040
*E. angulospora*	CBS 109906	Drinking water	Germany	JF747047
*E. angulospora*	CBS 482,92	Drinking water	Japan	JF747046
*E. castellanii*	CBS 110025	Drinking water	Germany	JF747072
*E. castellanii*	CBS 122325	Human, foot	Denmark	JF747068
*E. castellanii*	CBS 121496	Drinking water	Germany	JF747074
*E. castellanii*	CBS 109812	Drinking water	Germany	JF747075
*E. castellanii*	CBS 109914	drinking water	Germany	JF747076
*E. castellanii*	CBS 109915	Drinking water	Germany	JF747073
*E. castellanii*	CBS 158,58	Human, skin	Sri Lanka	JF747070

### DNA Extraction and Amplification

DNA extraction and quality tests were executed using glass beads (Sigma G9143) based on the methods described formerly [32]. DNA concentration and quality were tested spectrophotometrically at 260 and 280 nm (Shimadzu, Kyoto, Japan). ITS amplicons were produced with primers V9G and LS266 as described earlier [33]. PCR conditions were as follows: 95 °C for 5 min, followed by 35 cycles of 95 °C for 30 s, 55 °C for 30 s and 72 °C for 1 min, with final extension at 72 °C for 10 min. Amplification products were recognized by electrophoresis on 1% agarose gels.

### Padlock Probe Design

For the design of the RCA padlock probes, sequences of ITS regions of all tested *Exophiala* species, *Veronaea botryosa*, and closely related species from the CBS reference collection were aligned and adjusted manually using BioNumerics v. 4.61 (Applied Maths, St-Martens-Latem, Belgium) to identify informative nucleotide polymorphisms. Padlock probes targeting the ITS region were designed and were purchased from Invitrogen Inc. (Breda, the Netherlands). In order to optimize joining efficiency to target DNAs, the padlock probes were designed with minimal secondary structure and with Tm of the 5′ end probe binding arm near to or above ligation temperature (63 °C). To improve their discriminative specificity, the 3′-end binding arm was designed with a Tm 10–15 °C under ligation temperature. The linker part of each *Exophiala* species-specific probe obtained from [22] and the 5′- and the 3′-binding arms were designed in that article. Sequences of the both primers used for RCA and the oligonucleotide padlock probes are shown in Table 2. The oligonucleotide probes applied were c. 92–99 bp in length and contained two adjacent target complementary sequences (14–20 bp) with a spacer region (63 bp) to help loop formation and provide a template for RCA primer binding. Specificity of the probes was proved by BLAST examination in GenBank and in a validated database of filamentous fungi available at CBS for research purposes.

**Table 2:** Rolling circle amplification (RCA) padlock probes and padlock probe-specific primers used in this study

Probe or primer	Target species	Sequences and locations of the two binding arms in comparison with relevant reference
RCA1		5′-ATGGGCACCGAAGAAGCA-3′
RCA2		5′-CGCGCAGACACGATA-3′
Equi	***E. equina***	5′ P GGTTGGGCTACCGACGAGCG
		Gatca***TGCTTCTTCGGTGCCCAT****t*acgaggtgcggatagctac**CGCGCAGACACGATA**gtcta
		TRGTTAAAGATTTTAAT 3
Esal	***E. Salmonis***	5′ p AGGGGCCTCCACCAAACCGTC
		Gatca***TGCTTCTTCGGTGCCCAT****t*acgaggtgcggatagctac**CGCGCAGACACGATA**gtcta
		GGGGCAGATGCCCGCA 3′
Eopp	***E. opportunistica***	5′ p RAAGACCCCCCGGCGGTCCG
		Gatca***TGCTTCTTCGGTGCCCAT****t*acgaggtgcggatagctac**CGCGCAGACACGATA**gtcta
		GCGGGCCAAGGGGTRC 3′
Epis	***E. pisciphila***	5′ p AGACGGGCTCGCCGAAGCAAC
		Gatca***TGCTTCTTCGGTGCCCAT****t*acgaggtgcggatagctac**CGCGCAGACACGATA**gtcta
		CCCGGCGGTCCATTAC 3
Eagu	***E. aquamarina***	5′ p GGGGCGTCCACCAAGCCGTCCAA
		Gatca***TGCTTCTTCGGTGCCCAT****t*acgaggtgcggatagctac**CGCGCAGACACGATA**gtcta
		TGGACGCCCCGTGC 3′
Vbot	***V. botryosa***	5′ p CTGTTAGGGGTCCCCCGGCG
		Gatca***TGCTTCTTCGGTGCCCAT****t*acgaggtgcggatagctac**CGCGCAGACACGATA**gtcta
		GCGGGCCAGGAGACT 3′
Eang	***E. angulospora***	5′ p GACGGGCCCGCCGAAGCAAC
		Gatca***TGCTTCTTCGGTGCCCAT****t*acgaggtgcggatagctac**CGCGCAGACACGATA**gtcta
		CTCCGGCGGTCACGAA 3′
Ecas	***E. castellanii***	5′ p ACACCAAACCGTCCAACACCAA
		Gatca***TGCTTCTTCGGTGCCCAT****t*acgaggtgcggatagctac**CGCGCAGACACGATA**gtcta
		GGGGTGACGTTGCCG 3′

P: 5′-phosphorylation. Underlined: the binding arms of the padlock probes, which are joined by the backbone of the probe including the non-specific linker region. Bold: the binding region of the RCA1 and RCA2

### Ligation of Padlock Probe

One microliter of ITS amplicon was mixed with 2 U pfu DNA ligase (Epicentre Biotechnologies, Madison, WI, U.S.A.) and 0.1 μmol l^−1^ padlock probe in 20 mmol l^−1^ Tris–HCl (pH 7.5), 20 mmol l^−1^ Cl, 10 mmol l^−1^ MgCl_2_, 0.1% Igepal, 0.01 mmol l^−1^ rATP and 1 mmol l^−1^ DTT, with an overall total reaction volume of 10 μl. Padlock probe ligation reaction was performed as described by [34] by one denaturation cycle for 5 min at 94 °C, followed by five cycles of 30 s. at 94 °C and 4 min ligation at 63 °C.

### Exonucleolysis

Exonucleolysis is needed to eliminate unligated padlock probe and template PCR product and therefore minimize subsequent ligation-independent amplification events. It had been done in a 20-μl vol by addition of 10 U every one of exonuclease I and III (New England Biolabs, Hitchin, UK) to the ligation mixture and incubation at 37 °C for 30 min, followed by 94 °C for 3 min to inactive the exonuclease reaction.

### Rolling Circle Amplification (RCA) Reaction

Rolling circle amplification was executed in 50 µl reaction mixture containing 8 U *Bst* DNA polymerase (New England Biolabs), 400 μmol l^−1^ deoxynucleoside triphosphate mix and 10 pmol of every RCA primer in distilled water with 2 µl ligation product as template. Probe signals were amplified by incubation at 65 °C for 60 min, and accumulation of double stranded DNA products was visualized on a 1% agarose gel to validate the specificity of probe–template binding. Positive responses revealed a ladder-like structure, while negative responses showed a clean background. When the exonuclease step is omitted, some weak signal might be visible in gel electrophoresis [24].

## Results

**Fig. 1:**
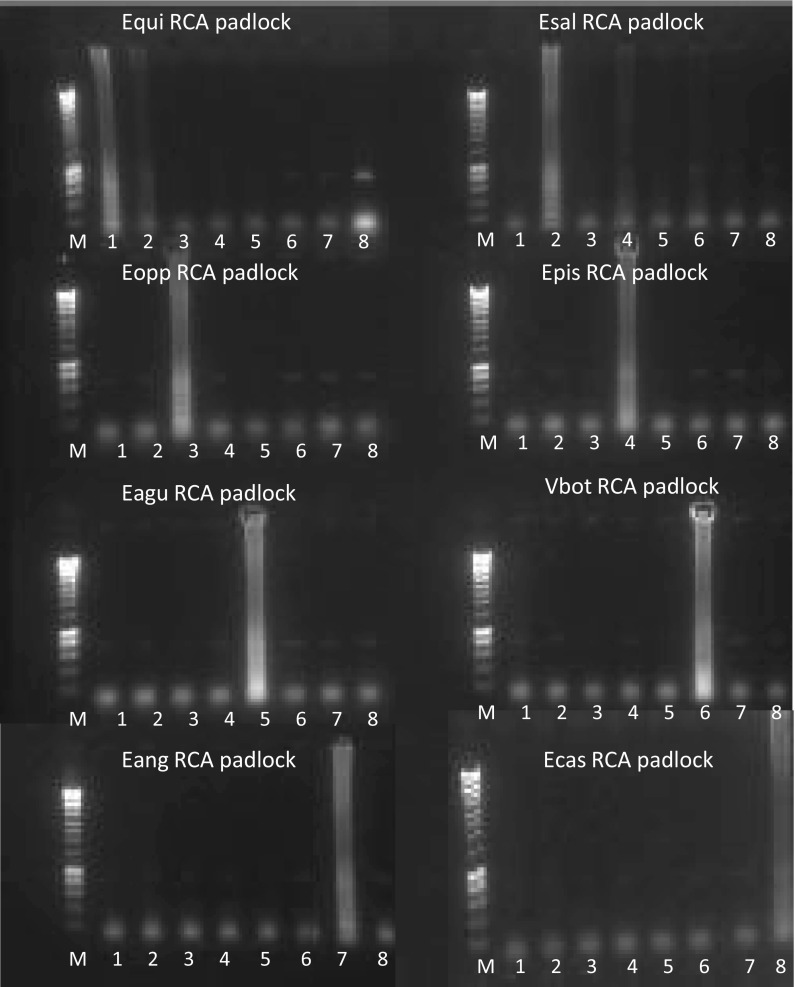
Proof of species specificity of RCA padlock probes and intraspecific variation of RCA response. Amplification and subsequent fluorescent banding were seen only with appropriate template–probe mixtures (Empty lanes denote the absence of signals with unmatched template–probe mixtures.) The species-specific probes are labeled as listed in Table 1 (Equi, *E. equina*; Esal, *E. Salmonis*; Eopp, *E. opportunistica*; Epis, *E. pisciphila*; Eagu, *E. aquamarina*; Vbot, *V. botryosa*; Eang, *E. angulospora*; Ecas, *E. castellanii*) lanes: M is 200-bp DNA MW marker (Eurogentec, the Netherlands); 1 to 8, RCA reaction with DNA of *E. equina* (CBS 109879) (lane 1), *E. Salmonis* (CBS 110371) (lane2), *E. opportunistica* (CBS 631.69) (lane 3), *E. pisciphila* (CBS 119913) (lane 4), *E. aquamarina* (CBS 119915) (lane 5), *V. botryosa* (CBS 121506) (lane 6), *E. angulospora* (CBS 119911) (lane 7) and *E. castellanii* (CBS 110025) (lane 8)

The general fungal primers ITS1 and ITS4 amplified the ITS location of all studied isolates. The ITS alignment revealed appropriate positions for the development of padlock probes that were able to differentiate between seven *Exophiala* species and the *Veronaea* out-group tested in this study. Each of the eight infectious species had several distinctive nucleotide positions. The duration of the RCA assay was 2 h. The tested species were unambiguously distinguished from each other and from different black yeast and relatives in the order *Chaetothyriales* compared by ITS sequence analysis and included as negative controls: the four closely related, clinically relevant species used for comparison, i.e., *Cladophialophora bantiana* (CBS 678.79), *Exophiala dermatitidis* (CBS 525.76), *E. bergeri* (CBS 526.76) and *Rhinocladiella mackenziei* (CBS 650.93) yielded negative results with the species-specific padlock probes (data not shown). Positive responses proved to be consisted and highly specific in all strains; all individual strains responded with respective probes being and were correctly identified as *Exophiala equina, E. salmonis*, *E. opportunistica, E. aquamarina, E. angulospora* and *E. castellanii*, as well as *Veronaea botryosa*, the nearest neighbor of the salmonis-clade [2]. No cross reaction was observed between any of the *Exophiala* species (Fig. 1). Products of the RCA responses were visualized by electrophoresis on 1% agarose gels. Positive reaction showed ladder-like patterns after RCA, while with negative results the background stayed clean. When the exonucleolysis step was deleted, a single poor band was apparent on the gel representing a non-specific band that did not interfere with the RCA reaction (data not shown). The concordance of RCA results and identification by multilocus sequencing was 100%.

## Discussion

Thermotolerance is generally considered as a prime condition for vertebrate pathogenicity. During the last decades, several black yeasts have been described in *Exophiala* that constantly lacked thermotolerance, but still were associated with animal disease, indicating that these fungi have other, intrinsic, temperature-independent infectious abilities [2]. Infections were especially within fish and amphibians, but sometimes also in invertebrates [14]. Such infections seem to be relatively regular, at least in captive and farmed fish and amphibians. Outbreaks in farmed and aquarium animals could cause serious losses to aquaculture and fishery industries [34], but because of the spread nature of reports it is hard to estimate the magnitude of the problem.

Rolling circle amplification is a powerful and easy, isothermal in vitro DNA amplification method emerging as a tool for quick detection of specific nucleic-acid sequences in DNA samples [35]. The use of a padlock probe to circularize oligonucleotides was produced by Nilsson [36]. The technique is on the basis of the replication of a short, single-stranded DNA circle by *Bst* DNA polymerases at constant temperature. Sequencing of the internal transcribed spacer (ITS) is the gold standard for species recognition of black yeast and relatives, as it provides sufficient resolution between species [18]. For analysis of large numbers of isolates in case of outbreaks and epidemiological monitoring, sequencing is nevertheless costly, time-consuming and impractical [22]. Furthermore, validated databases for comparison are needed, as GenBank information is polluted with wrongly identified sequences; we used a research database on black fungi housed at the Westerdijk Fungal Biodiversity Institute and of which a selection has been deposited in the ISHAM ITS Database (www.its.mycologylab.org). The RCA reaction is relatively free of requirement for high priced laboratory equipment and could be done within 2 h isothermally at 65 °C in a water bath, thermocycler, heat block or microwave. Nevertheless, positive signals are often visible 15 min after commencement of the RCA reaction when recognized by real-time PCR [19, 22]. The amplification product can be visualized by agarose gel electrophoresis, but can also be visualized in gel-free methods applying fluorescence staining of amplified product by SYBR Green in combination with a UV transilluminator. The progress of RCA probes to distinguish single species or groups of species depends on the presence of adequate sequence information and useful species-specific polymorphisms in genes of precisely identified species.

The objective of the current study was to begin a screening technique based on RCA for highly specific and rapid detection of waterborne *Exophiala* species which repeatedly occur in the form of outbreaks in farmed fish, enabling their unambiguous differentiation from related melanized fungi. The RCA method performed well elsewhere in the fungal Kingdom, e.g., in *Candida*, *Aspergillus*, *Scedosporium* [22], *Cryptococcus* [37], *Trichophyton* [19], *Fusarium* [21], human-pathogenic *Exophiala* [28], *Talaromyces marneffei* [24], *Scedosporium* [20], *Rhizopus* [23] and *Fonsecaea* [27]. A low-cost alternative to RCA might be loop-mediated isothermal amplification (LAMP). This technique uses a set of six oligonucleotide primers with eight joining sites hybridizing particularly to various parts of a target gene [38]. Najafzadeh et al. [27] compared RCA and LAMP detection for human-pathogenic *Fonsecaea* species and discovered that LAMP was extremely sensitive, but RCA become more specific.

In conclusion, RCA is a very fast (less than 1 working day), specific (down to the single-nucleotide level) and economical (no additional equipment required) method for specific and rapid identification of fungal pathogens where large numbers of strains need to be processed. Our results show a considerable potential of the method in the future in laboratories for fungal outbreak control, e.g., in farmed fish. The establishment of the test is relatively expensive, but with high throughput applications, the final result per strain will be rapid and cost-effective.
